# Metabolite localization by atmospheric pressure high-resolution scanning microprobe matrix-assisted laser desorption/ionization mass spectrometry imaging in whole-body sections and individual organs of the rove beetle *Paederus riparius*

**DOI:** 10.1007/s00216-014-8327-1

**Published:** 2014-11-26

**Authors:** Dhaka Ram Bhandari, Matthias Schott, Andreas Römpp, Andreas Vilcinskas, Bernhard Spengler

**Affiliations:** 1 Institute of Inorganic and Analytical Chemistry, Justus Liebig University Giessen, Schubertstraße 60, Building 16, 35392 Giessen, Germany; 2 Institute of Phytopathology and Applied Zoology, Justus Liebig University Giessen, Heinrich-Buff-Ring 26-32, 35392 Giessen, Germany

**Keywords:** *Paederus riparius*, Insects, Pederin, High-resolution mass spectrometry imaging, MALDI Imaging

## Abstract

Mass spectrometry imaging provides for non-targeted, label-free chemical imaging. In this study, atmospheric pressure high-resolution scanning microprobe matrix-assisted laser desorption/ionization mass spectrometry imaging (AP-SMALDI MSI) was used for the first time to describe the chemical distribution of the defensive compounds pederin, pseudopederin, and pederon in tissue sections (16 μm thick) of the rove beetle *Paederus riparius*. The whole-insect tissue section was scanned with a 20-μm step size. Mass resolution of the orbital trapping mass spectrometer was set to 100,000 at *m*/*z* 200. Additionally, organ-specific compounds were identified for brain, nerve cord, eggs, gut, ovaries, and malpighian tubules. To confirm the distribution of the specific compounds, individual organs from the insect were dissected, and MSI experiments were performed on the dissected organs. Three ganglia of the nerve cord, with a dimension of 250–500 μm, were measured with 10-μm spatial resolution. High-quality *m*/*z* images, based on high spatial resolution and high mass accuracy were generated. These features helped to assign mass spectral peaks with high confidence. Mass accuracy of the imaging experiments was <3 ppm root mean square error, and mapping of different compound classes from a single experiment was possible. This approach improved the understanding of the biochemistry of *P. riparius*. Concentration differences and distributions of pederin and its analogues could be visualized in the whole-insect section. Without any labeling, we assigned key lipids for specific organs to describe their location in the body and to identify morphological structures with a specificity higher than with staining or immunohistology methods.

**Electronic supplementary material:**

The online version of this article (doi:10.1007/s00216-014-8327-1) contains supplementary material, which is available to authorized users.

## Introduction

Insects are the most successful group of organisms on earth in terms of biodiversity. Their tremendous taxonomic and molecular diversity makes them indispensable as biological resources, and they are now being investigated as sources for natural products displaying pharmacological activities [1–3]. For example, insect-derived antimicrobial peptides or small molecules, exhibiting anti-infective activity, are explored as leads for the rational design of novel antibiotics [4–7].

One of the most potent substances is found in the ant-like beetle *Paederus riparius* [8]. This rove beetle of the Staphylinidae family is distributed worldwide. When the secretion from the *Paederus* beetle is exposed to the human skin, it causes dermatitis linearis, erythema, blisters (24 h after contact), and conjunctivitis [8–10]. The principal component accountable is pederin, initially characterized by processing 25 million individuals of *Paederus fuscipes* [8]. It has been revealed that the bacteria of *Pseudomonas* spp. are an endosymbiont within Paederinae producing pederin [11]. It has gained high research interest due to its potential anti-tumor activity [12]. In the insect, pederin acts most likely as a chemical defense agent, as it has been shown to protect beetle larvae against predatory spiders [13]. Chemically, pederin is a polyketide amide with two tetrahydropyran rings. It is known to be one of the most poisonous non-peptide compounds secreted by any insect [8]. The production, transportation, and accumulation of pederin in *Paederus* are still not fully understood. The bacterial symbiont which has been recognized to produce pederin cannot be cultured in the lab. The symbiont has an extremely reduced genome, and hence, it needs a specific surrounding which fulfills all its needs [11]. A new analytical technique is used here to achieve further insights into the tissue distribution of pederin and other metabolites within *P. riparius*.

The most commonly used analytical techniques for chemical analysis of insects are gas chromatography (GC), liquid chromatography (LC), and hyphenated techniques such as GC-mass spectrometry (MS) and LC-MS [14]. Most of these analytical techniques target specific molecules and include separation from the complex tissues. Consequently, the spatial information of the molecules is lost. This leads to the present fragmentary knowledge about the distribution of small molecules in insects. Visualizing the distribution of compounds can answer many physiological questions, by identifying the location for production, metabolization, and storage of compounds. In addition, the visualization of specific lipid distributions in insects can help to determine “marker” substances for specific organs [15–17].

In whole-body autoradiography [18], the molecule of interest is tagged with a radioactive compound, administered to the animal, and then its radioactivity is monitored in the body with spatial resolution. With this technology, it is not possible to distinguish tag-containing metabolites of the target molecules. In high-resolution microscopy-based immunochemistry, on the other hand, it is possible to image antigenic compounds only. The number of antigens available is limited and most of them are macromolecular. Thus, an alternative approach for imaging small molecules is needed. Chemical visualization of lipids in tissues is possible using staining by Nile Red (a lipid-soluble fluorescent dye) [19], Sudan Black [20], osmium (VIII) oxide [21], oil red O [22], and BODIPY 505/515 [23]. Most of these staining agents are used for neutral lipids, and, as with other staining techniques, individual lipid compounds cannot be distinguished. Thus, different lipid compounds cannot be visualized from a single tissue section. Visualizing organs and different tissues in insects is possible by vital staining, fluorescence microscopy, polarized light microscopy, histological staining, immunohistochemistry, and cytology, but none of these techniques give specific chemical information. One possibility is to perform microdissection for visualizing compounds in specific regions of the insect, but the extraction procedure limits the number and type of targeted compounds [24]. With the introduction of the label-free mass spectrometry imaging, such as matrix-assisted laser desorption/ionization (MALDI) [25], secondary ion mass spectrometry (SIMS) [26], desorption electrospray ionization (DESI) [27], laser ablation electrospray ionization (LAESI) [28], nanowire-assisted laser desorption ionization (NALDI) [29], nanostructure-initiator mass spectrometry (NIMS) [30], and liquid extraction surface analysis (LESA) [31], it is possible to obtain spatial information along with chemical identification of the visualized compounds. Not only the molecule of interest but simultaneously numerous other analytes can be detected and identified [32]. MALDI, DESI, and SIMS are the most widely used ionization techniques for MSI [33]. SIMS provides the highest spatial resolution, but the ionization of analytes requires vacuum, and due to the high energy of the ion beam, fragmentation of the analytes is severe. In contrast, DESI is a soft ionization technique, which operates under ambient conditions, but the spatial resolution is limited to 35 μm [33, 34]. MALDI, a soft ionization technique, is the most used imaging technique because of its inherent advantages, such as wide mass range, versatility, and directness of analysis. The disadvantage of MALDI with low-resolution instruments is interference of small molecules with matrix-derived background signals. This limitation can be overcome by using high-resolution and high mass accuracy mass analyzers such as Fourier transform ion cyclotron resonance (FT-ICR) or orbital trapping mass analyzers [35]. Also, elevated pressure in the ion source is known to reduce background signals from matrix, due to collisions with atmospheric gas [36, 37].

The other main limitation in MALDI imaging is the spatial resolution, which is limited to 30 to 50 μm in most of the commercially available imaging instruments. Combining an atmospheric pressure SMALDI imaging source (AP-SMALDI10®, TransMIT GmbH, Giessen, Germany) with an orbital trapping mass spectrometer (Q Exactive™ or Exactive™, Thermo Fisher Scientific GmbH, Bremen, Germany), a spatial resolution up to 3 μm has been obtained in a mouse brain tissue section. The analog instrumentation was employed in the following study. The source operates at atmospheric pressure (AP), preventing vaporization of the matrix. This additionally provides a great advantage of measuring the samples over longer periods of time [38], when images of large pixel number are to be acquired. Recently, MALDI imaging has been applied to understand the spatial distribution of various biomolecules such as lipids, proteins, peptides, drugs, and metabolites, showing a great prospect in the area of clinical sciences, medical diagnosis, and natural sciences [35, 38–48]. In contrast to mammalian tissue, only limited numbers of MS imaging experiments were reported for non-mammalian tissues. MS imaging of invertebrates tissue has been mainly focused on the invertebrate nervous system [49]. These imaging experiments were limited both in terms of spatial and mass resolution [50–58]. MALDI imaging of the surface of leaf-cutting ants (*Acromyrmex echinatior*) and a head section from the honeybee (*Apis mellifera*) has been reported at 100 and 50 μm of spatial resolution, respectively [59, 60]. MALDI TOF/TOF is the frequently used mass analyzer for imaging experiments. It provides the advantage of speed, wide mass range, and high sensitivity. Nevertheless, the mass resolution and mass accuracy of these instruments are inferior to those of FT-ICR and orbital trapping mass analyzers [61]. Using FT instruments, it is possible to identify compounds directly from tissue with higher confidence. Recently, high-resolution instruments have also been used to study neuropeptides from the central nervous system (CNS) of crustaceans (blue crabs) [57, 62]. Mapping of the chemical distribution in insects with high mass accuracy and high resolution is highly demanding, as the organs of insects are in the micrometer size range and are chemically very complex. In the following study, we applied for the first time AP-SMALDI MSI as a technique for whole-insect tissue imaging in order to visualize the tissue and organ distribution of pederin and other metabolites in *P. riparius*.

## Materials and methods

### Chemicals

Trifluoroacetic acid (TFA), water (HPLC grade), and 2,5-dihydroxybenzoic acid (DHB) were purchased from Fluka (Neu Ulm, Germany), tragacanth from Sigma-Aldrich (Steinheim, Germany), and acetone from Merck (Darmstadt, Germany).

### Insect and its organs

*P. riparius* were field collected near Bayreuth, Germany. Specimens were frozen at −20 °C prior to sample preparation. To obtain uniform thin sections, the whole insect was embedded in 10 % tragacanth gum, followed by preparation of the frozen block at −80 °C. These blocks were sliced to a thickness of 16 μm using a cryrostat (HM 525 cryostat, Thermo Scientific, Dreieich, Germany). The embedding material was removed carefully with a painting brush, preventing the distortion of tissue. Then the section was thaw mounted on a microscope glass slide (ground edges frosted, VWR international GmbH, Darmstadt, Germany). The samples were brought to room temperature in a desiccator to avoid condensation of humidity. An optical image of the section was generated with an Olympus BX-40 microscope (Olympus Europa GmbH, Hamburg, Germany).

Internal organs from the insect were dissected manually and then placed on a microscope glass slide. The thickness and uniformity of the samples were measured with a microscope (Olympus BX-40). A dedicated matrix preparation system (SMALDIPrep, TransMIT GmbH, Giessen, Germany) was used to spray the matrix solution of 30 mg/ml of 2,5-dihydroxybenzoic acid in 50:50 (*v*/*v*) acetone:H_2_O (0.1 % TFA) on top of the tissue sections [63]. The homogeneity and the crystal sizes were controlled after matrix application by microscopy before fixing the sample on the sample holder of the imaging source.

### Instrumentation

Mass spectra were generated using a Fourier transform orbital trapping mass spectrometer (Exactive™ or Q Exactive™, Thermo Scientific GmbH, Bremen, Germany) coupled to an atmospheric pressure scanning microprobe matrix-assisted laser desorption/ionization imaging source (AP-SMALDI10®, TransMIT GmbH, Giessen, Germany) [64]. For desorption/ionization of the analyte, a nitrogen laser with a repetition rate of 60 Hz and wavelength of 337 nm was used. The laser beam was focused to an ablation spot diameter of approx. 5 μm [65] using a centrally bored objective lens. The samples were scanned by the movement of the x-, y-, and z-stages placed in front of the transfer capillary to the mass spectrometer with different step sizes (10 to 20 μm) for different samples. Oversampling by the laser spot size was avoided during the scanning of the tissue. The target voltage was set to 4.3 kV, and it was operated in positive-ion mode. Automatic gain control was set fixed to 500-ms C-trap opening time (“injection time”). Internal calibration was achieved by using lock masses. The cycle time for one pixel at 100,000 mass resolving power was 1.1 s for a *m*/*z* range of 100–1000.

### Data processing

Ion images of selected *m*/*z* values were generated using the MIRION software package [66] with a bin width of ∆*m*/*z* = ±5 ppm [66]. The ion images were scaled to the highest intensity for each substance separately. RGB images of three different *m*/*z* values were overlaid and displayed simultaneously. Compounds were assigned based on high mass accuracy (root mean square error <3 ppm). For the analysis of tandem mass spectra, Mass Frontier™ version 7.0 (Thermo Fisher Scientific GmbH, Bremen, Germany) was used [67].

## Results

**Fig. 1:**
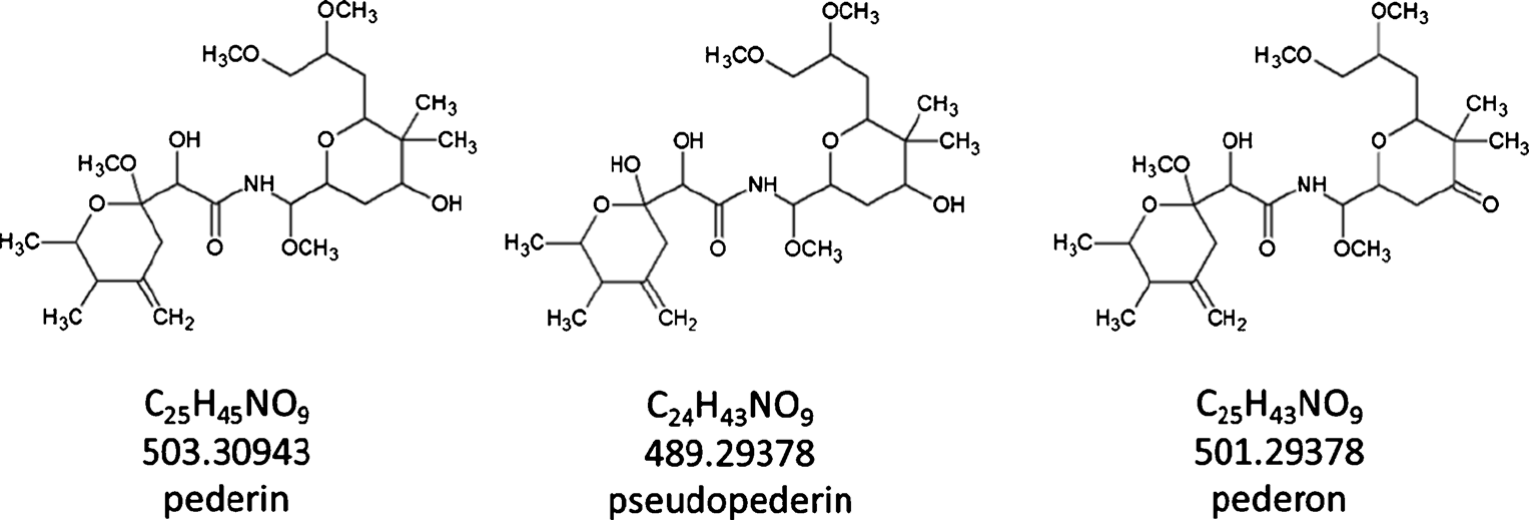
Structure of pederin, pseudopederin, and pederon

**Fig. 2:**
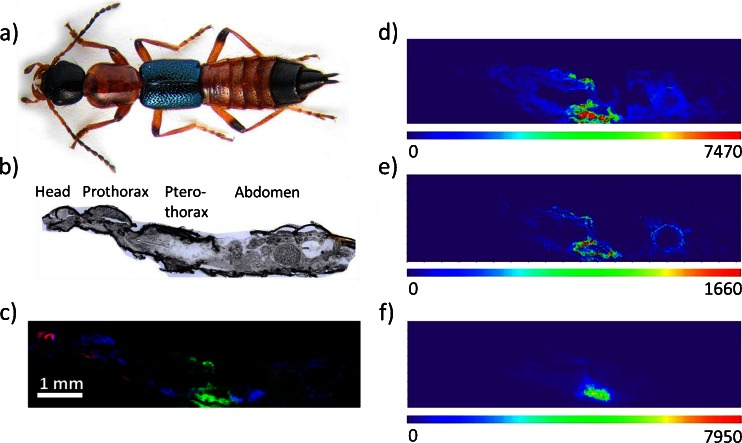
Whole-insect AP-SMALDI mass spectrometry imaging of *Paederus riparius*. **a** Photographic image of the insect [68]. **b** Optical image of a 16-μm-thick sagittal section of the insect. **c** Red-green-blue (RGB) ion images: *green*, pederin [M + Na]^+^, *m*/*z* 526.29865, prominent in the fourth abdominal segment, location of the reservoir of the defensive gland; *blue*, [PC(38:6) + K]^+^, *m*/*z* 844.52531 whole body; *red*, [PS(42:5) + K]^+^, *m*/*z* 904.54644. **d**–**f** Pseudocolor images of pederin, pseudopederin, and norspermine in the whole-insect section. Each image (**d**–**e**) represents the sum ion signals from sodium-attached and potassium-attached molecules of pederin and pseudopederin; **f** pseudocolor image of norspermine, [M + H]^+^, *m*/*z* 189.20747. Mass values used for generation of images are given in Table 1 (exact masses). Each image was created for an exact *m*/*z* value with a bin with ±5 ppm for measured centroid *m*/*z* values. Images were scaled to the highest signal intensity per image. Color scale bar values below the scales refer to minimum (*violet*) and maximum (*red*) signal intensities. MS image sizes are 385 × 100 pixels. Step size was set to 20 μm

- Imaging of whole insects
  Sectioning of the whole insect is relatively complicated compared to sectioning of individual organs or mammalian tissues. The exoskeleton (cuticle) of insects functions as a primary barrier against pathogens, parasites, and attacks from predators. This stiff outer structure makes the sectioning procedure complicated, as the hard shell tends to shift the softer haemocoel and thereby to destroy the actual location and content of the organs. Suitable embedding materials, like tragacanth, can assist to obtain thin uniform sections from the whole insect. Generally, embedding is used to stabilize tissues before sectioning. The hard solid block in which the tissue is embedded assists the microtomy process by preventing shrinkage, distortion, and loss of cellular constituents. In case of insect microtomy, the embedding material’s main task is to assist alignment, to provide support to the exoskeleton and to prevent distortions generated during sectioning. Compared to the more common MALDI embedding material carboxymethylcellulose (CMC), tragacanth prevents the floating of the sample during the embedding process. After a thin uniform section (16 μm) was obtained, MALDI matrix was applied to the sample. The whole-insect MALDI-MS imaging experiment was performed on an area of 7700 × 2000 μm^2^ with a step size of 20 μm in positive-ion mode in a mass range of *m*/*z* 150–1000. The resolving power of the MS was set up to 100,000 at *m*/*z* 200. Then the molecules of interest, pederin, pseudopederin, and pederon (Fig. 1) were imaged. The *m*/*z* images were generated from the raw data without normalization. Two modes of generating colored images were used in this study. First, the “RGB mode” was used to compare the distribution of three different specific substances within the measurement. Here, each measured ion signal was correlated to the intensity of one of the basic colors: red, green, and blue. Second, a “rainbow pseudocolor” table was used to improve the dynamic visualization range and visibility of the distribution of a single compound in the measurement. The rainbow color table is shown in Fig. 2d–f (pederin, pseudopederin, and norspermine). Figure 2a is a photographic image of the insect [68]. Figure 2b shows photographic image of a 16-μm thick sagittal section of the whole insect before matrix coating. The MS image in Fig. 2c shows a RGB image of pederin [M + Na]^+^, *m*/*z* 526.29865 in green and two phospholipids in red and blue. The substance pederin can most prominently be found in the abdominal region of the fourth segment where the reservoir of this defensive compound is located [13]. The MS image corresponds to the optical image in Fig. 2b that helps to morphologically assign the spatial distribution of the defensive compounds. The MS image in Fig. 2c also exhibits the distribution of a phosphatidylcholine (PC) in blue [PC(38:6) + K]^+^, *m*/*z* 844.52531, distributed throughout the whole body, and a phosphatidylserine (PS) in red [PS(42:5) + K]^+^, *m*/*z* 904.54644, localized in the brain and the nerve cord. The toxin pseudopederin and pederon have similar but distinguishable distributions as represented in Fig. 2e and in Electronic Supplementary Material (ESM) Fig. S1. The concentrations of these substances significantly differ in the insect. Among these compounds, pederon has the lowest concentration in the insect. In addition to pederin and its analogues, the polyamine norspermine (*m*/*z* 189.20737, [M + H]^+^) was found and imaged in the defensive gland (Fig. 2f) of the insect. To the author’s best knowledge, this compound is reported here for the first time as being present in *P. riparius* (Table 1).

**Table 1:** Exact monomolecular ion mass values used for generation of the images in Fig. 2

Compound	Molecular formula	Adduct	Calculated exact mass	Measured accurate mass
Pederin	C_25_H_45_NO_9_	[M + Na]^+^	526.29865	526.29895
	C_25_H_45_NO_9_	[M + K]^+^	542.27259	542.27294
Pseudopederin	C_24_H_43_NO_9_	[M + Na]^+^	512.28300	512.28332
	C_24_H_43_NO_9_	[M + K]^+^	528.25694	528.25727
Norspermine	C_9_H_24_N_4_	[M + H]^+^	189.20747	189.20753
PC(38:6)	C_46_H_80_NO_8_P	[M + K]^+^	844.52531	844.52577
PS(42:5)	C_48_H_84_NO_10_P	[M + K]^+^	904.54644	904.54673

**Fig. 3:**
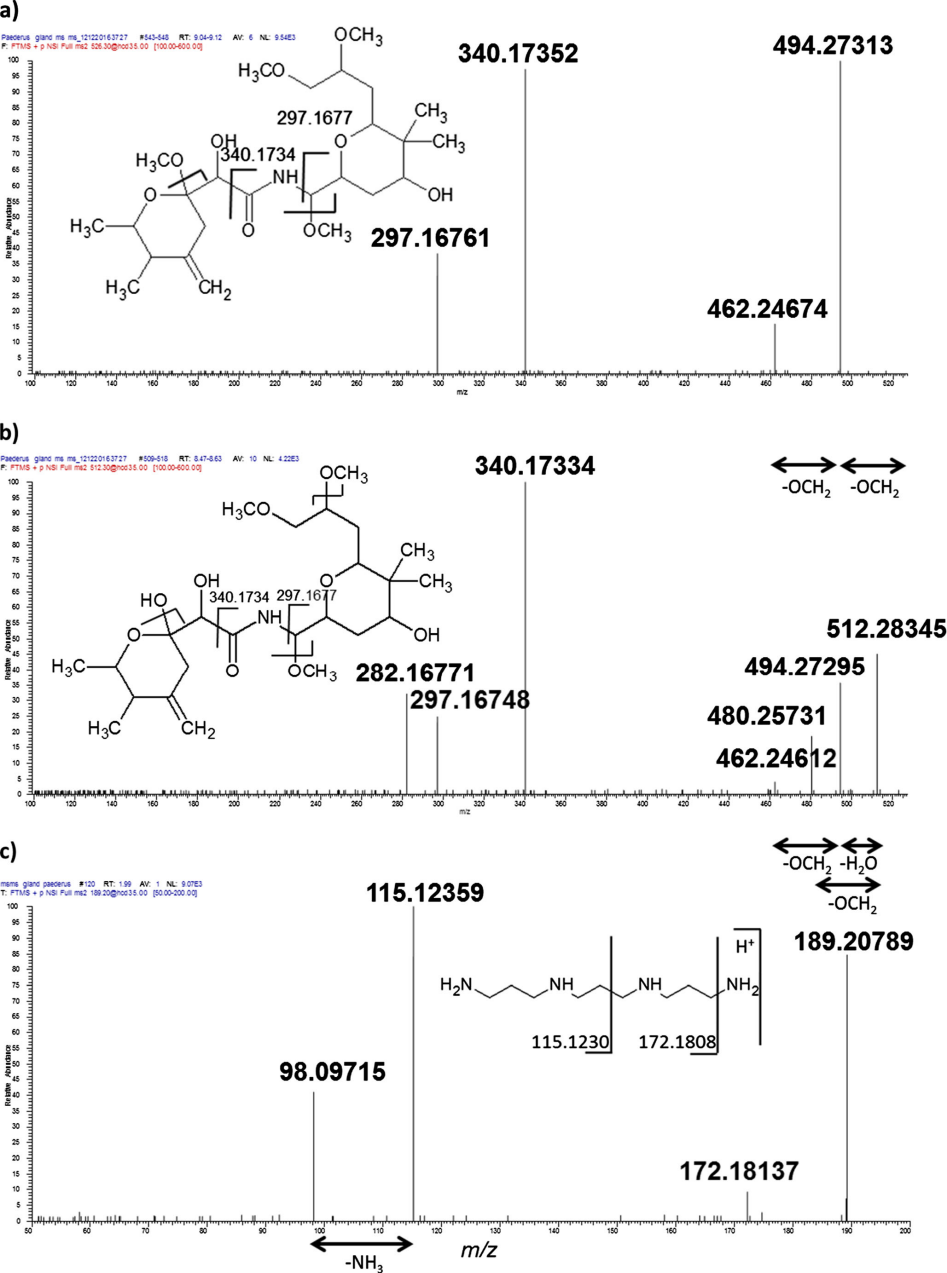
On-tissue MS/MS spectra: **a** MS/MS spectrum of pederin *m*/*z* 526.29865 [M + Na]^+^, averaged over all pixels; **b** pseudopederin precursor *m*/*z* 512.28300, [M + Na]^+^, averaged over all pixels; and **c** single pixel MS/MS spectrum of norspermine *m*/*z* = 189.20737 [M + H]^+^. Observed fragment ion *m*/*z* values are indicated in the presumed fragmentation scheme

**Fig. 4:**
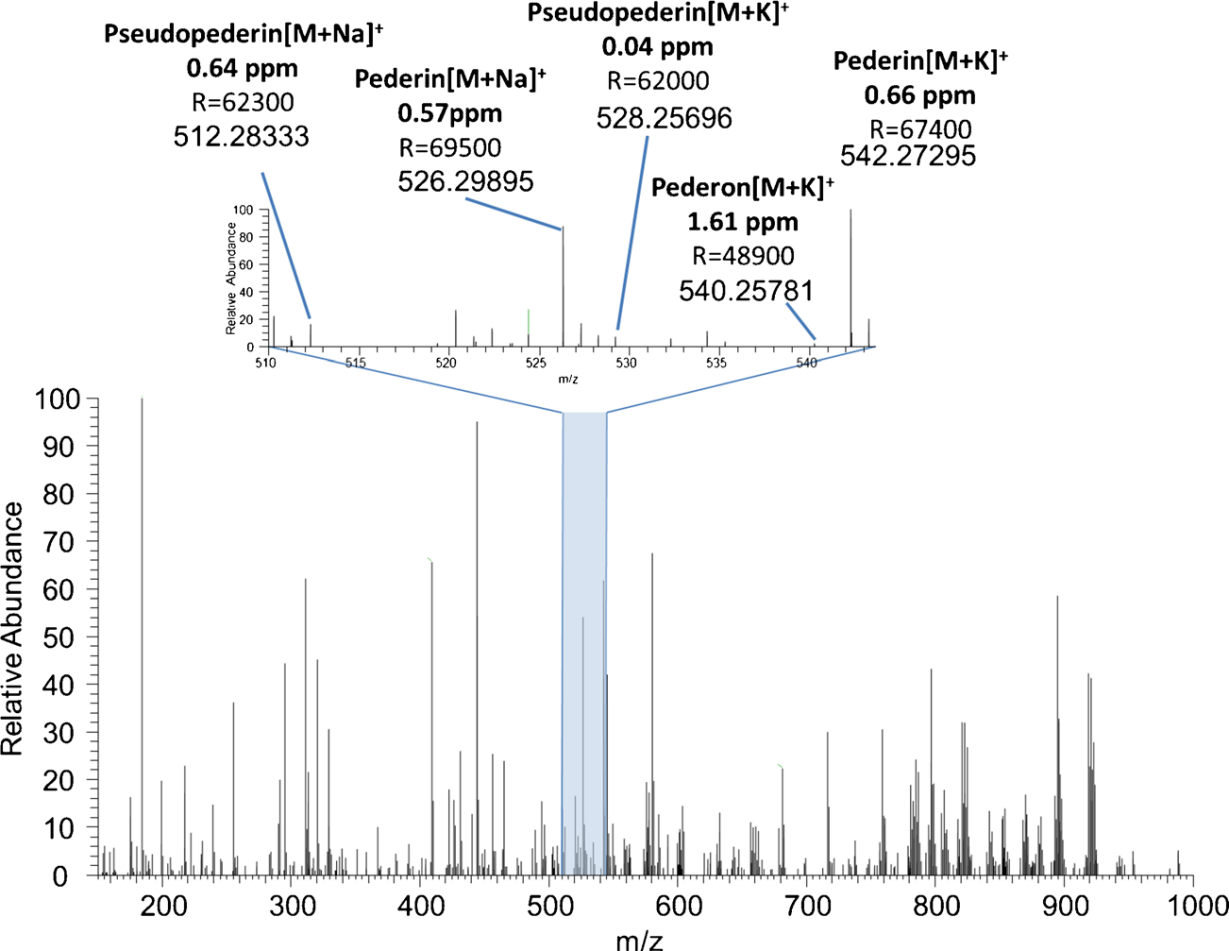
AP-SMALDI MS spectrum obtained from a single 5-μm spot measured with a 20-μm step size, showing the toxins pederin, pseudopederin, and pederon. The mass resolution (*R*) for the whole-body imaging was set to 100,000 at *m*/*z* 200. Deviations between calculated and measured *m*/*z* values are indicated for labeled peaks

**Fig. 5:**
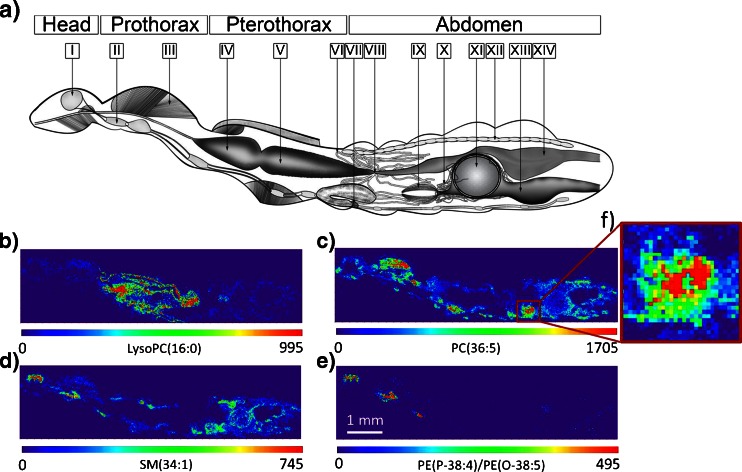
Compounds exhibiting different distributions in the insect body section: **a** Graphical representation of the location of organs in *P. riparius*: *I*, brain; *II*, nerve cord; *III*, muscle; *IV*, crop; *V*, midgut; *VI*, malpighian tubules; *VII*, sternal gland with attached reservoir; *VIII*, pylorus; *IX*, ovaries; *X*, accessory glands; *XI*, egg; *XII*, heart; *XIII*, oviduct; and *XIV*, rectum. **b**–**e** AP-SMALDI images of ±5 ppm bin width exhibiting different distributions of compounds listed in Table 2. **f** The pixel structure (correlated with the imaging step size) of the image in a zoomed-in region of ovaries. Images were scaled to the highest signal intensity per image. Color scale bar values refer to minimum (*violet*) and maximum (*red*) signal intensities. Pixel size is 20 μm × 20 μm

(b) On-tissue MS/MS
  In order to provide a higher confidence in identification of pederin, pseudopederin, and norspermine, on-tissue MS/MS was performed using a Q Exactive (Thermo Scientific GmbH, Bremen, Germany) mass spectrometer. Figure 3a–c shows the MS/MS spectra of pederin, pseudopederin, and norspermine, respectively. A MS/MS isolation window of 0.4 Da was used for the selection of the precursor ion. Same higher-energy collisional dissociation (HCD) energy was used for tandem MS. As pederin is more labile to fragmentation than pseudopederin and norspermine, the precursor peak of pederin is missing in the spectra. Fragment ions of the precursor pseudopederin (*m*/*z* 512.28300), sampled from the gland region of the tissue, resemble those of the precursor pederin (*m*/*z* 526.29865), indicating that they are structurally related. Nevertheless, the fragment peak at *m*/*z* 480.25741, which is obtained only from the precursor pseudopederin, indicates the difference between the structures. The software Mass Frontier™ version 7.0 was used to generate possible fragment ions from pederin and pseudopederin. The theoretical fragments generated by the software were identical to the observed fragments in our experiment. It was additionally confirmed that pseudopederin is not an MS-generated in-source fragmentation product of pederin, based on the observed different LC retention times of pseudopederin and pederin obtained from the homogenate extract of an insect and based on the lack of pseudopederin signal-increase in an in-source CID experiment of pederin. The assignment of norspermine was also supported by tandem mass spectrometry (Fig. 3c). Further experiments using standards are required to confirm the structure of the compound.

(c) Visualization of metabolites in the whole-insect section
  In addition to the metabolites mentioned above, many other peaks could be putatively assigned from a single experiment along with the spatial information (ESM Table S1 and Fig. S2). The assignment was based on high mass accuracy, <3 ppm root mean square error (RMSE) (ESM Table S1), literature [11, 13, 69–77], and Metlin database [78]. Figure 4 shows a mass spectrum obtained from a single spot of approx. 5 μm in diameter, measured with an imaging step size of 20 μm, resulting in MS images with a pixel size of 20 × 20 μm^2^. The spectrum is zoomed to the *m*/*z* range of the molecules of interest. Various groups of compounds, glycerophospholipids, sphingolipids, glycerolipids, carnitines, acetyl carnitines, fatty acids, glycerophosphocholines, and nucleic acid derivatives were assigned by accurate mass and visualized (ESM Table S1). Figure 5a represents an anatomical drawing for locating the organs in the 16-μm thick section of *P. riparius* from Fig. 2b. Through MS imaging of a whole-insect body, it is possible to locate different organs through organ-specific metabolites. Figure 5 exhibits ion images of different compound classes such as lysoPC(16:0) (Fig. 5b) prominent in the pylorus, crop, and the midgut; PC(36:5) (Fig. 5c), distributed uniformly but with higher intensity in the muscle region; sphingomyelin, SM(34:1) (Fig. 5d), localized in the region of the brain, nerve cord, and fat body; and phosphatidylethanolamine, PE(P-38:4)/PE(O-38:5) (Fig. 5e), occurring mainly in the brain and nerve cord region. Figure 5f is a zoomed-in view of Fig. 5c, in the region of the ovaries, exhibiting differentiable individual pixels, which are correlated with the step size of the tissue imaging data acquisition. The images were generated using a *m*/*z* bin width of ±5 ppm relative to exact mass values (Table 2). Images were scaled to the highest signal intensity per image. Color scale bar values refer to the minimum (violet) and maximum (red) signal intensities. Pixel size is 20 μm × 20 μm.

**Table 2:** Theoretical monoisotopic ion mass values of selected lipids from different lipid classes

Compound	Molecular formula	Adduct	Calculated exact mass	Measured accurate mass
LysoPC(16:0)	C_24_H_50_NO_7_P	[M + H]^+^	496.33977	496.34001
		[M + Na]^+^	518.32171	518.32202
		[M + K]^+^	534.29565	534.29599
PC(36:5)	C_44_H_78_NO_8_P	[M + Na]^+^	802.53573	802.53607
		[M + K]^+^	818.50966	818.51004
		[M + H]^+^	703.57485	703.57519
SM(34:1)	C_39_H_79_N_2_O_6_P	[M + Na]^+^	725.55680	725.55714
		[M + K]^+^	741.53073	741.53138
		[M + H]^+^	752.55887	752.55757
PE(P-38:4)/PE(O-38:5)	C_43_H_78_NO_7_P	[M + Na]^+^	774.54081	774.54102
		[M + K]^+^	790.51475	790.51462
  For lipids, the ion intensities of protonated, sodium-attached, and potassium-attached signals were summed up to generate images. The mass values used for generation of images are shown in Table 2.
  The different regions of the insects are specific to certain masses. The images were generated with exact mass of individual ±5 ppm. Images were scaled to the highest signal intensity per image. Color scale bar values refer to minimum (violet) and maximum (red) signal intensities. Pixel size is 20 μm × 20 μm.

**Fig. 6:**
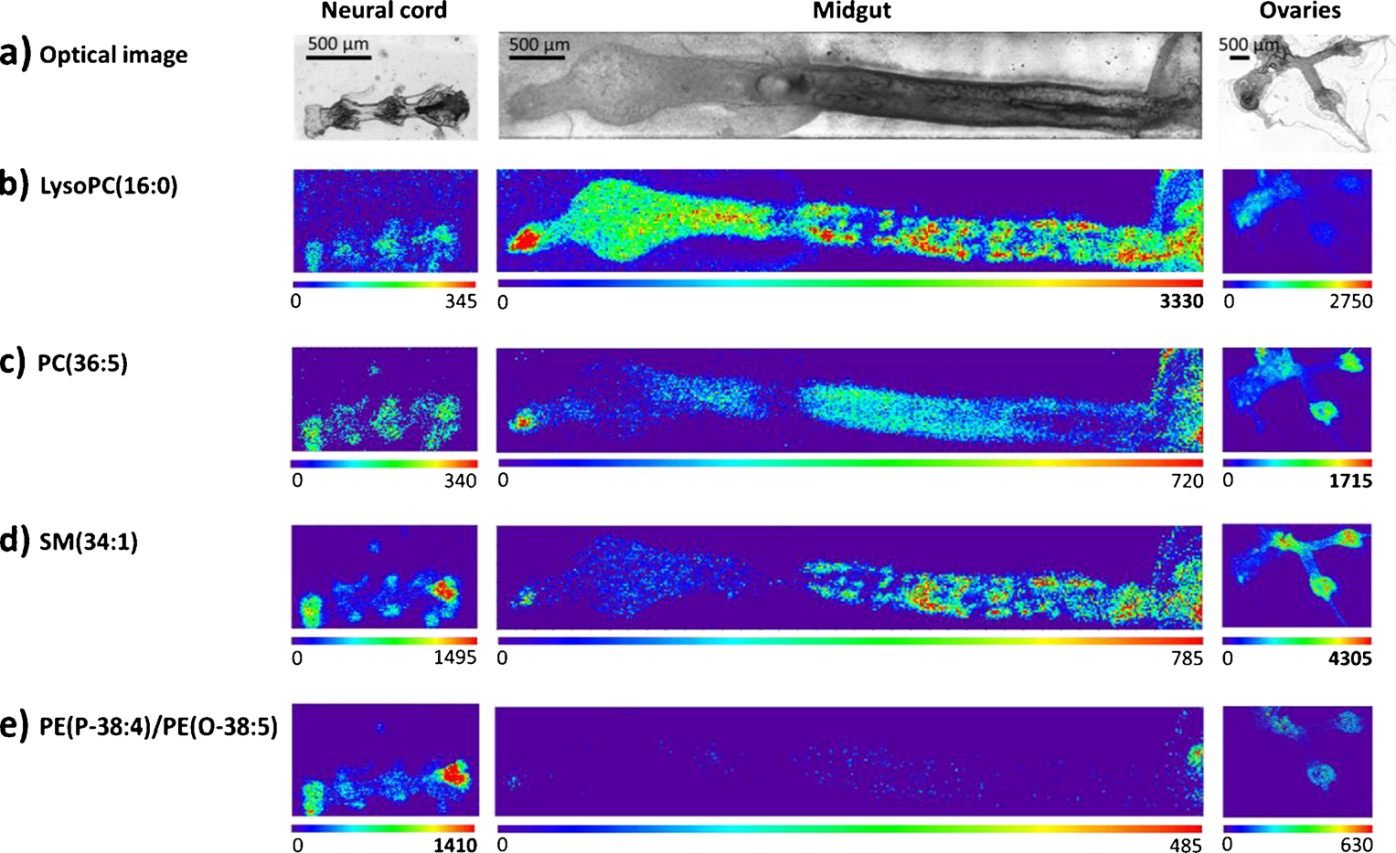
AP-SMALDI images of three dissected organs, using selected *m*/*z* signals as in Fig. 5**a** optical images of dissected nerve cord, midgut, and ovary (*left to right*). **b**–**e** *m*/*z* images that exhibit different distributions in different organs. The *m*/*z* images were generated using exact mass values (Table 2) with a bin width of ±5 ppm. Pixel size was 10 μm (*neural cord*) and 15 μm (*gut and ovaries*). Images were scaled to the highest signal intensity per image. Color scale bar values refer to minimum (*violet*) and maximum (*red*) signal intensities

**Fig. 7:**
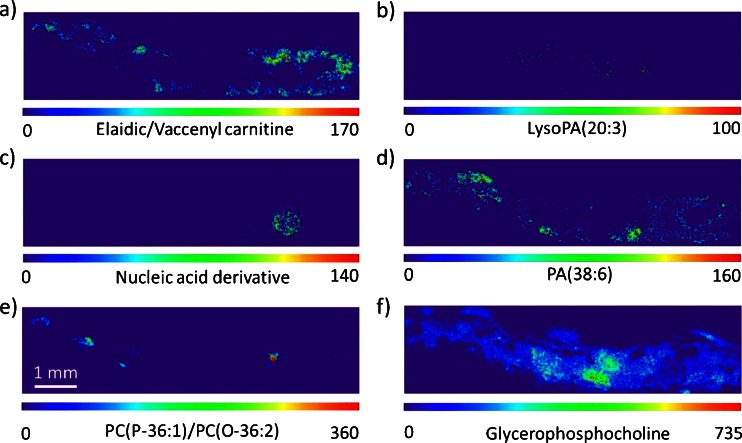
AP-SMALDI images of compounds from Table 3: Compounds were found to have specific distributions in different regions of the insect. Images were generated using the exact ion mass of ±5 ppm. Images were scaled to the highest signal intensity per image. Color scale bar values refer to minimum (*violet*) and maximum (*red*) signal intensities

(d) Imaging of internal organs from *P. riparius*
  Assignment of organs in a histological section of an insect is difficult, as even with staining methods different tissues exhibit similar colors and are therefore hardly discernible. We proved the specificity of masses to a particular organ by measuring the corresponding dissected organs with SMALDI imaging. In Fig. 5, specific compounds exhibit abundance variations in different areas. This correlates with a higher abundance of molecules in a specific organ. Even the ventral nerve cord, a fine structure which originates from the insects’ brain and continuous ventral with several ganglia, each having a diameter of around 250 μm, can be visualized by high-resolution SMALDI imaging in the whole-body section. To prove the identity of these organs, ovary, nerve cord, and gut were dissected and selected for additional MS imaging experiments. Before matrix application, the organs were transferred onto a glass slide and the uniformity of each sample was checked before measurements. In Fig. 6, a comparison of the different signals imaged in Fig. 5 for three organs, neural cord, midgut, and ovaries, is shown. Figure 6a shows the optical image of the nerve cord, midgut, and ovaries before the measurement. LysoPC(16:0) was measured with higher intensity in the midgut region in the whole-body imaging (Fig. 5b), also exhibiting higher intensity in the measurement of the dissected midgut (Fig. 6b, note max scale bar values of images). Likewise, PC(36:5) was found in higher intensity in the ovary measurement (Fig. 6c), as expected from Fig. 5c. SM(34:1) was especially present in the ovaries and the nerve cord (Fig. 6d). PE(P-34:3)/PE(O-38:5) was observed with the highest intensities in the brain and nerve cord in the whole-insect measurement (Fig. 5e), confirmed in the measurement of the dissected part of the nerve cord (Fig. 6e).

(e) Visualization of other metabolites
  In addition to the metabolites mentioned above, Fig. 7 shows other compound classes that exhibit specific distributions across the insect body. Elaidic/vaccenyl carnitine is mainly distributed in the hindgut, muscle, and fat body. LysoPA (lysophosphatidic acids) have a similar distribution as lysoPC(16:0) in the pylorus and part of the hindgut. Nucleic acid derivatives are highly concentrated in the egg. The phosphatidic acid, PA(38:6), is distributed rather uniformly in the insect, but high intensities were found in the area of muscles. PC(P-36:1)/PC(O-36:2) exhibits a localization similar to the ether lipid PE(P-38:4)/PE(O-38:5), mainly found in the brain and the nerve cord and also in a region close to the egg, which we could not assign to a specific organ. Glycerophosphocholine, which acts as an osmotic agent, was found to be present at higher concentration in the malpighian tubules of the insect (Table 3) [79].

**Table 3:** Theoretical monoisotopic masses used for generation of the images in Fig. 7

Compound	Molecular formula	Adduct	Calculated exact mass	Measured accurate mass
Elaidic/vaccenyl carnitine	C_25_H_47_NO_4_	[M + H]^+^	426.35779	426.35784
LysoPA(20:3)	C_23_H_41_O_7_P	[M + K]^+^	499.22215	499.22227
Nucleic acid: adenosine triphosphate	C_10_H_16_N_5_O_13_P_3_	[M + H]^+^	508.00302	508.00335
PA(38:6)	C_41_H_69_O_8_P	[M + K]^+^	759.43616	759.43614
PC(P-36:1)/PC(O-36:2)	C_44_H_86_NO_7_P	[M + H]^+^	772.62147	772.62176
Glycerophosphocholine	C_8_H_20_NO_6_P	[M + K]^+^	296.06598	296.06599

## Discussion

Mass spectrometry imaging has the potential to complement the classical tissue visualizing methods such as histochemistry, histopathology, fluorescence microscopy with labeled antibodies, and autoradiography. Its main advantage lies in the possibility to identify different chemical compound classes and individual compounds in a single experiment without labeling [38, 43, 80]. This leads to the possibility to assign different tissues by distinct marker substances which express a higher concentration in a specific organ [81]. For insects, only a small number of MS imaging experiments were reported [54, 56, 62], performed on the surface of the insect [56, 59] or in a specific organ [62].

Here instead, a sagittal longitudinal whole-insect tissue section was scanned with a 20-μm step size by AP-SMALDI MSI. Internal organs could be assigned, and the defensive compounds of the rove beetle could be localized without labeling. Several substances were assigned by high-resolution MSI. The distribution of pederin and its analogues has been selected as a target for tissue analysis with high resolution in mass and space, to obtain insights into the biological pathway of pederin synthesis and metabolism. The distribution of pederin and its analogues is roughly understood from dissection experiments. Here, we show the distribution of these substances in situ by performing whole-insect imaging. This will help to explain the localization and transportation of these molecules through the insect in further studies. The capability of high mass resolution, high mass accuracy, and on-tissue MS/MS provided evidence in the structural identification of pederin and pseudopederin in the tissue (Fig. 3). For the first time, norspermine was detected in the glandular reservoir of *P. riparius* defensive gland. As norspermine was found in our study in a high concentration in close neighborhood to pederin, it might serve as an additional toxin as it is reported in spiders [82, 83]. Another explanation is that it plays a role in the transportation of pederin in cells or inside of *P. riparius* [84]. It has been reported that polyamines can be used as vectors for anti-cancer drugs, in utilizing the polyamine pathway to carry potent toxins inside cells [85, 86].

Additionally, we could identify several mass signals as being organ-specific in *P. riparius*. It has to be tested in future experiments, if these mass signals are conserved and can be used for characterization of other target insects. Hankin et al. showed within a singular class of lipids and for similar tissue types that the intensity of lipid molecular ion signals, generated in positive-ion MALDI MSI experiments, does in fact relate to the quantity of molecules in a tissue sample [87]. We were able to clearly assign mass signals to different morphological compartments. The following *P. riparius* markers for specific organs were reported here for the first time: The brain and nerve system were represented by *m*/*z* 752.55887, corresponding to PE(P-38:4)/PE(O-38:5). These compounds were also reported in mammalian nerve cells [88–90] and seem to be highly conserved across species. The digestive system was well-represented by lysoPC(16:0). Additionally, the sodium-attached and potassium-attached species of this molecule were observed with similar distribution, supporting the assignment based on accurate mass. LysoPC isomers were reported in *Spotoptera litoralis* larvae [91]. Phosphatidylcholine PC(36:5) was found to be highly concentrated in insect muscles, especially in the musculature of the dorsal part of the prothorax and the leg musculature in the ventral part of the pterothorax. It was also found to be highly concentrated in the ovaries. The compound has been reported in insect embryonic tissue where large amounts of nutrients are stored for independent survival [92–94]. Sphingomyelin SM(34:1) (Figs. 5d and 6d) exhibits a high concentration in the insects’ brain and ventral nerve cord. This substance can also be seen in much lower concentration in all abdominal organs except the midgut. Sphingomyelin compounds were reported from the sheath of invertebrate nerve fibers [73]. Similar to SM(34:1) as mentioned above, PE(P-38:4)/PE(O-38:5) shows a high concentration almost exclusively in the brain and nerve cord.

Based on MSI of dissected organs with 10- and 15-μm step sizes (Fig. 6), the molecular representation and actual location of these organs in the whole insect and the marker substances in Fig. 5 were confirmed. Figure 6 reveals that the distribution of these substances is inhomogeneous in the substructure of these individual organs.

Other compound classes were also visualized in specific organs (Fig. 7). Elaidic carnitine and vaccenyl carnitine cannot be distinguished based on exact mass as the difference is only in the position of the double bond, as in the case of PE(P-38:4)/PE(O-38:5) and PC(P-36:1)/PC(O-36:2). These metabolites (carnitines) are known to be part of the carnitine shuttle which helps to transfer lipids to the mitochondria where the oxidation of long-chain fatty acids takes place [95]. In Fig. 7a, the elaidic carnitine/vaccenyl carnitine distributions are shown to be enhanced in the fat body and muscle tissue where fatty acid metabolism is active. LysoPA shows a distribution similar to lysoPC, i.e., being enhanced in the midgut region. LysoPA and lysoPC compounds contain one fatty acid per lipid molecule in position sn-1. LysoPC is produced by hydrolysis of dietary and biliary phosphatidylcholine in mammals and is absorbed as such in the intestines [96]. Phosphatidic acid (PA), an important intermediate in lipid biosynthesis, was detected as sodium-attached or potassium-attached molecules (ESM Table S1) with a distribution primarily in the muscles and fat body (Fig. 7d). In the egg region of the insect, a higher concentration of nucleic acid derivatives compared to the rest of the tissue was detected (Fig. 7c, ESM Fig. S2) with mass differences of the phosphate groups, indicating the presence of nucleoside monophosphate, nucleoside diphosphate, and nucleoside triphosphate at *m*/*z* 348.07036 [M + H]^+^, 428.03669 [M + H]^+^, and 508.00302 [M + H]^+^, respectively (ESM Fig. S2 and Table S1). The enhanced presence of these compounds might indicate a higher cellular activity in the egg of the insect. PC(P-36:1)/PC(O-36:2), an ether lipid with a choline head group, also shows a distribution in the brain and nerves, similar to the ether lipid PE(P-38:4)/PE(O-38:5), except for a small region near the egg. At the transition of the mid- to hindgut, the malpighian tubules are connected. This region is characterized by the sodium-attached glycerophosphocholine (*m*/*z* 280.0920), which also acts as an osmotic agent in other organisms [79, 97]. Malphigian tubules function as an excretory and osmoregulatory system in insects.

## Conclusion

High-resolution AP-SMALDI-MSI of a whole insect revealed chemical morphology data and detailed tissue distributions of natural products in the rove beetle. We have visualized the tissue distribution of pederin and other natural products in an unprecedented, high spatial resolution, thus introducing the applicability of this molecular imaging technique for the analysis of insects and other small animals. The additional information provided by individual-organ imaging confirmed the organ identification in the whole-insect measurement. This is a novel way of characterizing insect organs and of visualizing metabolites in a single insect section. In this experiment, pederin analogues, pseudopederin and pederon, were identified and mapped simultaneously, exhibiting a similar distribution as pederin. Norspermine, a polyamine, was reported for the first time in *P. riparius*. The data obtained from the AP-SMALDI imaging experiments can be used as a location library in future investigations of the metabolome of *P. riparius*.

## Electronic supplementary material

Below is the link to the electronic supplementary material.

ESM 1 (PDF 782 kb)
