# Supplementary material for: Metabolite localization by atmospheric pressure high-resolution scanning microprobe matrix-assisted laser desorption/ionization mass spectrometry imaging in whole-body sections and individual organs of the rove beetle Paederus riparius
Source: Anal Bioanal Chem. 2014 Nov 26;407(8):2189–201. doi: 10.1007/s00216-014-8327-1 (PMC4357651; doi:10.1007/s00216-014-8327-1)
Supplement: Supplementary file 1 — (PDF 782 kb) [file 216_2014_8327_MOESM1_ESM.pdf]

Analytical and Bioanalytical Chemistry

Electronic Supplementary Material

Metabolite localization by atmospheric pressure high-resolution scanning microprobe matrix-assisted laser desorption/ionization mass spectrometry imaging in whole-body sections and individual organs of the rove beetle *Paederus riparius*

Dhaka Ram Bhandari, Matthias Schott, Andreas Römpp, Andreas Vilcinskas, Bernhard Spengler

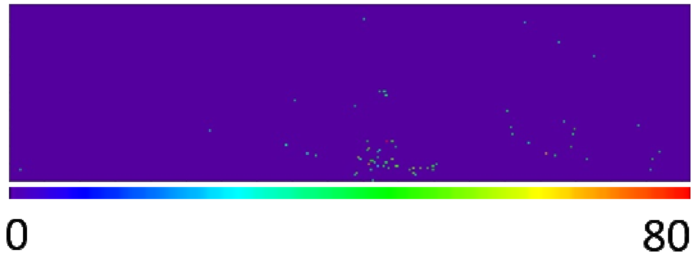

Fig. S1 AP-SMALDI *m/z* image of [pederon+K]+, *m/z* 540.25694, present in low quantity in the insect. MS image 385x100 pixels; 20 µm step size; bin width ± 5 ppm

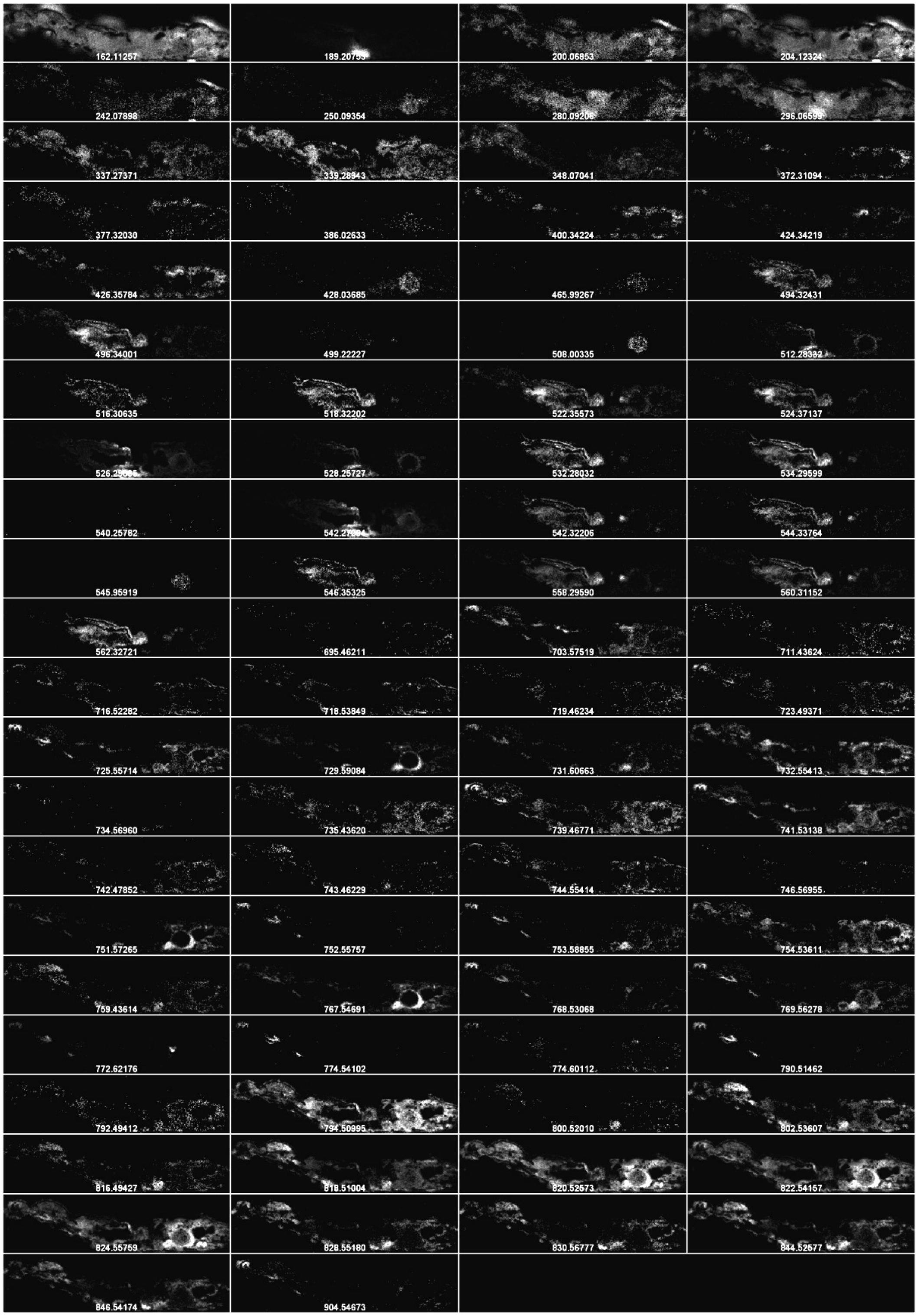

Fig. S2 AP-SMALDI *m/z* images of compounds listed in Table S1

Table S1 List of peak putatively identified compounds from a single MALDI imaging experiment, based on high mass accuracy (< 3 ppm, root mean error). Organs have been identified by comparative MALDI imaging experiments on dissected organs and comparative histological morphological studies

	**Compound**	**Molecular formula**	**ion detected**	**Calculated exact mass**	**Measured accurate mass (intensity weighted mean value over 10 ppm bin width)**	**Mass error of mean (10 ppm bin width) / ppm**	**RMS error (10 ppm bin width)**	**Distribution of the compound in the whole insect**
1	sterol derivative	$C_{28}H_{42}O$	$[M+H-H_2O]^+$	377.32028	377.32030	0.05	1.21	Haemolymph
2	LysoPC(16:0)	$C_{24}H_{50}NO_7P$	$[M+H]^+$	496.33977	496.34001	0.48	0.96	Most prominent in crop, also midgut and less in thoracical fat body
3	LysoPC(16:0)	$C_{24}H_{50}NO_7P$	$[M+Na]^+$	518.32171	518.32202	0.60	1.38	Crop, Pylorus area and less in thoracical fat body
4	LysoPC(16:0)	$C_{24}H_{50}NO_7P$	$[M+K]^+$	534.29620	534.29599	-0.39	0.87	Most prominent in Pylorus area, also crop and midgut
5	LysoPC(16:1)	$C_{24}H_{48}NO_7P$	$[M+H]^+$	494.32412	494.32431	0.38	1.01	Most prominent at crop, also pylorus and midgut
6	LysoPC(16:1)	$C_{24}H_{48}NO_7P$	$[M+Na]^+$	516.30606	516.30635	0.56	1.38	Most prominent in Pylorus area, also crop and midgut
7	LysoPC(16:1)	$C_{24}H_{48}NO_7P$	$[M+K]^+$	532.28000	532.28032	0.60	1.19	Most prominent in Pylorus area, also crop and midgut
8	LysoPC(18:0)	$C_{26}H_{54}NO_7P$	$[M+H]^+$	524.37107	524.37137	0.57	1.04	Most prominent at crop, also pylorus and midgut
9	LysoPC(18:0)	$C_{26}H_{54}NO_7P$	$[M+Na]^+$	546.35301	546.35325	0.44	1.18	Most prominent at crop, also pylorus and midgut
10	LysoPC(18:0)	$C_{26}H_{54}NO_7P$	$[M+K]^+$	562.32695	562.32721	0.46	0.99	Midgut
11	LysoPC(18:1)	$C_{26}H_{52}NO_7P$	$[M+H]^+$	522.35542	522.35573	0.59	1.08	Most prominent at crop, also pylorus and midgut and part of hindgut
12	LysoPC(18:1)	$C_{26}H_{52}NO_7P$	$[M+Na]^+$	544.33736	544.33764	0.51	1.27	Midgut and part of hindgut

13	LysoPC(18:1)	$C_{26}H_{52}NO_7P$	$[M+K]^+$	560.31130	560.31152	0.39	0.99	Pylorus and part of hindgut
14	LysoPC(18:2)	$C_{26}H_{50}NO_7P$	$[M+Na]^+$	542.32171	542.32206	0.65	1.31	Pylorus and part of hindgut
15	LysoPC(18:2)	$C_{26}H_{50}NO_7P$	$[M+K]^+$	558.29565	558.29590	0.45	0.95	Pylorus and part of hindgut
16	PC(30:1)	$C_{38}H_{74}NO_8P$	$[M+K]^+$	742.47836	742.47852	0.22	1.66	Fat body
17	PC(32:0)	$C_{40}H_{80}NO_8P$	$[M+H]^+$	734.56943	734.56960	0.23	2.02	Nerve cord
18	PC(32:1)	$C_{40}H_{78}NO_8P$	$[M+H]^+$	732.55378	732.55413	0.48	1.18	Muscles and fat body
19	PC(32:1)	$C_{40}H_{78}NO_8P$	$[M+Na]^+$	754.53573	754.53611	0.50	1.52	Muscles and fat body
20	PC(34:3)	$C_{42}H_{78}NO_8P$	$[M+K]^+$	794.50966	794.50995	0.37	0.86	Haemolymph, most prominent in crop and ovaries
21	PC(34:4)	$C_{42}H_{76}NO_8P$	$[M+K]^+$	792.49401	792.49412	0.14	1.61	Haemolymph
22	PC(36:2)	$C_{44}H_{84}NO_8P$	$[M+K]^+$	824.55661	824.55759	1.19	1.42	Ovarial accessory glands oviduct
23	PC(36:3)	$C_{44}H_{82}NO_8P$	$[M+K]^+$	822.54096	822.54157	0.74	0.98	Ovarial accessory glands oviduct
24	PC(36:4)	$C_{44}H_{80}NO_8P$	$[M+K]^+$	820.52531	820.52573	0.51	0.85	Ovarial accessory glands oviduct, muscles
25	PC(36:5)	$C_{44}H_{78}NO_8P$	$[M+Na]^+$	802.53573	802.53607	0.42	1.19	Muscles, Ovaries
26	PC(36:5)	$C_{44}H_{78}NO_8P$	$[M+K]^+$	818.50966	818.51004	0.46	0.97	Muscles, Ovaries
27	PC(36:6)	$C_{44}H_{76}NO_8P$	$[M+Na]^+$	800.52008	800.52010	0.02	1.68	Ovaries
28	PC(36:6)	$C_{44}H_{76}NO_8P$	$[M+K]^+$	816.49401	816.49427	0.32	1.45	Muscles, Ovaries
29	PC(38:5)	$C_{46}H_{82}NO_8P$	$[M+Na]^+$	830.56703	830.56777	0.89	1.58	Sternal organ between defensive gland an ovaries
30	PC(38:5)	$C_{46}H_{82}NO_8P$	$[M+K]^+$	846.54096	846.54174	0.92	1.45	Sternal organ between defensive gland an ovaries
31	PC(38:6)	$C_{46}H_{80}NO_8P$	$[M+Na]^+$	828.55138	828.55180	0.51	1.30	Muscles Ovaries, unknown abdominal region
32	PC(38:6)	$C_{46}H_{80}NO_8P$	$[M+K]^+$	844.52531	844.52577	0.54	1.15	Muscles Ovaries, Sternal organ between defensive gland and ovaries
33	PC(P-36:1)	$C_{44}H_{86}NO_7P$	$[M+H]^+$	772.62147	772.62176	0.38	1.33	Brain and neural cord, Part of oviduct
34	PE(34:1)	$C_{39}H_{76}NO_8P$	$[M+H]^+$	718.53813	718.53849	0.50	1.34	Cuticle

35	PE(34:2)	$C_{39}H_{74}NO_8P$	$[M+H]^+$	716.52248	716.52282	0.47	1.50	Cuticle
36	PE(36:1)	$C_{41}H_{80}NO_8P$	$[M+H]^+$	746.56943	746.56955	0.16	1.99	Cuticle
37	PE(36:2)	$C_{41}H_{78}NO_8P$	$[M+H]^+$	744.55378	744.55414	0.48	1.51	Cuticle, haemolymph
38	PE(38:1)	$C_{43}H_{84}NO_8P$	$[M+H]^+$	774.60073	774.60112	0.50	1.81	Brain, nerve cord
39	PE(P-36:1)	$C_{41}H_{80}NO_7P$	$[M+K]^+$	768.53040	768.53068	0.36	1.48	Brain, nerve cord
40	PE(P-38:4)	$C_{43}H_{78}NO_7P$	$[M+K]^+$	790.51475	790.51462	-0.16	1.43	Brain, nerve cord
41	PE(P-38:4)/PE(O-38:5)	$C_{43}H_{78}NO_7P$	$[M+H]^+$	752.55887	752.55757	-1.73	2.45	Brain, nerve cord
42	PE(P-38:4)/PE(O-38:5)	$C_{43}H_{78}NO_7P$	$[M+Na]^+$	774.54081	774.54102	0.27	1.52	Brain, nerve cord
43	LysoPA(20:3)	$C_{23}H_{41}O_7P$	$[M+K]^+$	499.22215	499.22227	0.24	1.45	Pylorus and part of hindgut
44	PA(34:2)	$C_{37}H_{69}O_8P$	$[M+Na]^+$	695.46223	695.46211	-0.17	1.87	Haemolyph, low density
45	PA(34:2)	$C_{37}H_{69}O_8P$	$[M+K]^+$	711.43616	711.43624	0.11	1.60	Haemolymph
46	PA(36:2)	$C_{39}H_{73}O_8P$	$[M+Na]^+$	723.49353	723.49371	0.25	1.53	Nerve cord, Haemolyph
47	PA(36:2)	$C_{39}H_{73}O_8P$	$[M+K]^+$	739.46746	739.46771	0.34	1.32	Nerve cord, Haemolyph
48	PA(36:4)	$C_{39}H_{69}O_8P$	$[M+Na]^+$	719.46223	719.46234	0.15	1.66	Haemolyph,
49	PA(36:4)	$C_{39}H_{69}O_8P$	$[M+K]^+$	735.43616	735.43620	0.05	1.53	Haemolymph
50	PA(38:6)	$C_{41}H_{69}O_8P$	$[M+Na]^+$	743.46223	743.46229	0.08	1.79	Muscles, Ovaries, low density
51	PA(38:6)	$C_{41}H_{69}O_8P$	$[M+K]^+$	759.43616	759.43614	-0.03	1.53	Muscles, Ovaries
52	PS(42:5)	$C_{48}H_{84}NO_{10}P$	$[M+K]^+$	904.54644	904.54673	0.32	1.55	Brain, nerv cord
53	SM(34:1)	$C_{39}H_{79}N_2O_6P$	$[M+H]^+$	703.57485	703.57519	0.48	1.30	Brain, nerve cord, upper Pterothorax
54	SM(34:1)	$C_{39}H_{79}N_2O_6P$	$[M+Na]^+$	725.55680	725.55714	0.47	1.35	Brain, nerve cord, fat body
55	SM(34:1)	$C_{39}H_{79}N_2O_6P$	$[M+K]^+$	741.53073	741.53138	0.88	1.53	Brain, nerve cord, fat body, ovaries
56	SM(36:1)	$C_{41}H_{83}N_2O_6P$	$[M+H]^+$	731.60615	731.60663	0.66	1.49	Brain, nerve cord, left part of accessory glands
57	SM(36:1)	$C_{41}H_{83}N_2O_6P$	$[M+Na]^+$	753.58810	753.58855	0.60	1.51	Brain, nerve cord, left part of accessory glands

58	SM(36:1)	$C_{41}H_{83}N_2O_6P$	$[M+K]^+$	769.56258	769.56278	0.26	1.39	Brain, nerve cord, left part of accessory glands
59	SM(36:2)	$C_{41}H_{81}N_2O_6P$	$[M+H]^+$	729.59050	729.59084	0.47	1.19	Ovarial accessory glands, and sternal defensive gland reservoir
60	SM(36:2)	$C_{41}H_{81}N_2O_6P$	$[M+Na]^+$	751.57245	751.57265	0.27	1.07	Ovarial accessory glands, sternal defensive gland reservoir, nerve cord
61	SM(36:2)	$C_{41}H_{81}N_2O_6P$	$[M+K]^+$	767.54638	767.54691	0.69	1.32	Ovarial accessory glands, sternal defensive gland reservoir
62	MG(18:1)	$C_{21}H_{40}O_4$	$[M+H-H_2O]^+$	339.28937	339.28943	0.18	0.98	Leaking of compounds
63	MG(18:2)	$C_{21}H_{38}O_4$	$[M+H-H_2O]^+$	337.27372	337.27371	-0.03	1.04	Haemolymph
64	Acetylcarnitine	$C_9H_{17}NO_4$	$[M+H]^+$	204.12303	204.12324	1.03	1.10	Leaking of compounds
65	Acetylcarnitine	$C_9H_{17}NO_4$	$[M+K]^+$	242.07892	242.07898	0.25	0.92	Leaking of compounds
66	Carnitine	$C_7H_{15}NO_3$	$[M+H]^+$	162.11247	162.11257	0.62	0.73	Leaking of compounds
67	Carnitine	$C_7H_{15}NO_3$	$[M+K]^+$	200.06835	200.06853	0.90	1.07	Leaking of compounds
68	Carnitine:-Elaidic carnitine/Vaccenyl carnitine	$C_{25}H_{47}NO_4$	$[M+H]^+$	426.35779	426.35784	0.12	0.95	Hindgut
69	Carnitine:-Linoleyl carnitine/Linoelaidyl carnitine	$C_{25}H_{45}NO_4$	$[M+H]^+$	424.34214	424.34219	0.12	1.15	Hindgut
70	Carnitine:-Palmitoylcarnitine	$C_{23}H_{45}NO_4$	$[M+H]^+$	400.34214	400.34224	0.25	1.09	Fat body
71	Carnitine:-Tetradecanoylcarnitine	$C_{21}H_{41}NO_4$	$[M+H]^+$	372.31084	372.31094	0.27	1.12	Fat body
72	Glycerophosphocholine	$C_8H_{20}NO_6P$	$[M+Na]^+$	280.09204	280.09206	0.07	0.91	Malpighian tubules
73	Glycerophosphocholine	$C_8H_{20}NO_6P$	$[M+K]^+$	296.06598	296.06599	0.03	0.71	Malpighian tubules
74	Nucleic acid:Adenosine/Deoxyguanosine	$C_{10}H_{13}N_5O_4$	$[M+H-H_2O]^+$	250.09347	250.09354	0.28	1.00	Ovaries, egg
75	Nucleic acid:Adenosine 3\',5\'-bisphosphate (PAP)/Deoxyguanosine diphosphate (dGDP)	$C_{10}H_{15}N_5O_{10}P_2$	$[M+H]^+$	428.03669	428.03685	0.37	1.09	Ovaries, egg
76	Nucleic acid:Adenosine monophosphate (PAP)/Deoxyguanosine	$C_{10}H_{14}N_5O_7P$	$[M+H]^+$	348.07036	348.07041	0.14	1.02	Nerve cord, egg

	monophosphate (dGDP)							
77	Nucleic acid:Adenosine monophosphate (PAP)	$C_{10}H_{14}N_5O_7P$	$[M+K]^+$	386.02624	386.02633	0.23	1.55	low intensity ovaries and muscles
78	Nucleic acid:Deoxyguanosine diphosphate (dGDP)	$C_{10}H_{15}N_5O_{10}P_2$	$[M+K]^+$	465.99257	465.99267	0.21	1.59	low intensity ovaries and egg
79	Nucleic acid:Adenosine triphosphate	$C_{10}H_{16}N_5O_{13}P_3$	$[M+H]^+$	508.00302	508.00335	0.65	1.69	Egg
80	Nucleic acid:Adenosine triphosphate	$C_{10}H_{16}N_5O_{13}P_3$	$[M+K]^+$	545.95890	545.95919	0.52	2.21	Egg
81	Pederin	$C_{25}H_{45}NO_9$	$[M+Na]^+$	526.29865	526.29895	0.57	0.94	Sternal defensive gland reservoir
82	Pederin	$C_{25}H_{45}NO_9$	$[M+K]^+$	542.27259	542.27294	0.65	0.96	Sternal defensive gland reservoir
83	Pseudopederin	$C_{24}H_{43}NO_9$	$[M+Na]^+$	512.28300	512.28332	0.62	1.21	Sternal defensive gland reservoir
84	Pseudopederin	$C_{24}H_{43}NO_9$	$[M+K]^+$	528.25694	528.25727	0.62	1.16	Sternal defensive gland reservoir, egg shell
85	Pederon	$C_{25}H_{43}NO_9$	$[M+K]^+$	540.25694	540.25782	1.63	2.79	Sternal defensive gland reservoir, egg shell
86	Norspermine	$C_9H_{24}N_4$	[M+H]+	189.20737	189.20753	0.85	1.21	Sternal defensive gland reservoir
